# Transcutaneous Auricular Vagus Nerve Stimulation Modulates Performance but Not Pupil Size During Nonnative Speech Category Learning

**DOI:** 10.1044/2023_JSLHR-22-00596

**Published:** 2023-08-31

**Authors:** Jacie R. McHaney, William L. Schuerman, Matthew K. Leonard, Bharath Chandrasekaran

**Affiliations:** a Northwestern University, Evanston, IL; b University of California San Francisco

## Abstract

**Purpose:**

Subthreshold transcutaneous auricular vagus nerve stimulation (taVNS) synchronized with behavioral training can selectively enhance nonnative speech category learning in adults. Prior work has demonstrated that behavioral performance increases when taVNS is paired with easier-to-learn Mandarin tone categories in native English listeners, relative to when taVNS is paired with harder-to-learn Mandarin tone categories or without taVNS. Mechanistically, this temporally precise plasticity has been attributed to noradrenergic modulation. However, prior work did not specifically utilize methodologies that indexed noradrenergic modulation and, therefore, was unable to explicitly test this hypothesis. Our goal for this study was to use pupillometry to gain mechanistic insights into taVNS behavioral effects.

**Method:**

Thirty-eight participants learned to categorize Mandarin tones while pupillometry was recorded. In a double-blinded design, participants were divided into two taVNS groups that, as in the prior study, differed according to whether taVNS was paired with easier-to-learn tones or harder-to-learn tones. Learning performance and pupillary responses were measured using linear mixed-effects models.

**Results:**

We found that taVNS did not have any tone-specific or group behavioral or pupillary effects. However, in an exploratory analysis, we observed that taVNS did lead to faster rates of learning on trials paired with stimulation, particularly for those who were stimulated at lower amplitudes.

**Conclusions:**

Our results suggest that pupillary responses may not be a reliable marker of locus coeruleus–norepinephrine system activity in humans. However, future research should systematically examine the effects of stimulation amplitude on both behavior and pupillary responses.

**Supplemental Material:**

https://doi.org/10.23641/asha.24036666

Vagus nerve stimulation (VNS) is emerging as a promising technique for modulating neuroplasticity and cognition (Vonck et al., 2014). VNS has been used as a treatment for conditions, such as epilepsy (Ben-Menachem, 2002) and Alzheimer's disease (Merrill et al., 2006), and as an adjuvant to stroke rehabilitation (Engineer et al., 2019). Traditionally, VNS requires surgical implantation of electrodes at the cervical branch in the neck, and therefore, its therapeutic reach is not widely accessible to the general population. Recently, transcutaneous auricular VNS (taVNS) has gained attention as a potential inexpensive and accessible alternative to implanted VNS. This technique uses surface electrodes to target the auricular branch of the vagus nerve, which runs near the surface of the skin in the outer ear, innervating the tragus, cymba concha, and cymba cavum (Badran, Dowdle, et al., 2018; Urbin et al., 2021). Preliminary research suggests that taVNS, within certain parameters, may elicit effects comparable to traditional implanted VNS without the need for surgery (Schuerman et al., 2021; Yakunina et al., 2017). However, there is a need to identify a reliable biomarker of taVNS efficacy to further advance taVNS applications for both clinical and nonclinical purposes.

Building on extensive VNS studies in animal models (Borland et al., 2016; Buell et al., 2019; Clark et al., 1995, 1998), taVNS has been used to enhance plasticity in the mature, adult brain. In previous studies examining the effects of taVNS on Mandarin tone category learning in native English-speaking adults, tVNS enhanced overall learning when subthreshold tVNS was paired with specific Mandarin tones (Llanos et al., 2020) and improved learning to a greater extent compared to a sham stimulation control group (Pandža et al., 2020). Mandarin has four tone categories that differ in their pitch patterns. As compared to nontonal languages such as English, these pitch patterns are linguistically relevant at the syllable level and can differentiate word meaning in Mandarin, which often makes it difficult for native English listeners to discern. Mandarin tones are composed of high-level (Tone 1), low-rising (Tone 2), low-dipping (Tone 3), and high-falling (Tone 4) tones that primarily vary in their relative pitch (i.e., high vs. low; Tone 1 and Tone 3) and pitch change (i.e., rising vs. falling; Tone 2 and Tone 4) acoustic dimensions (see Figure 1A).

**Figure 1.**
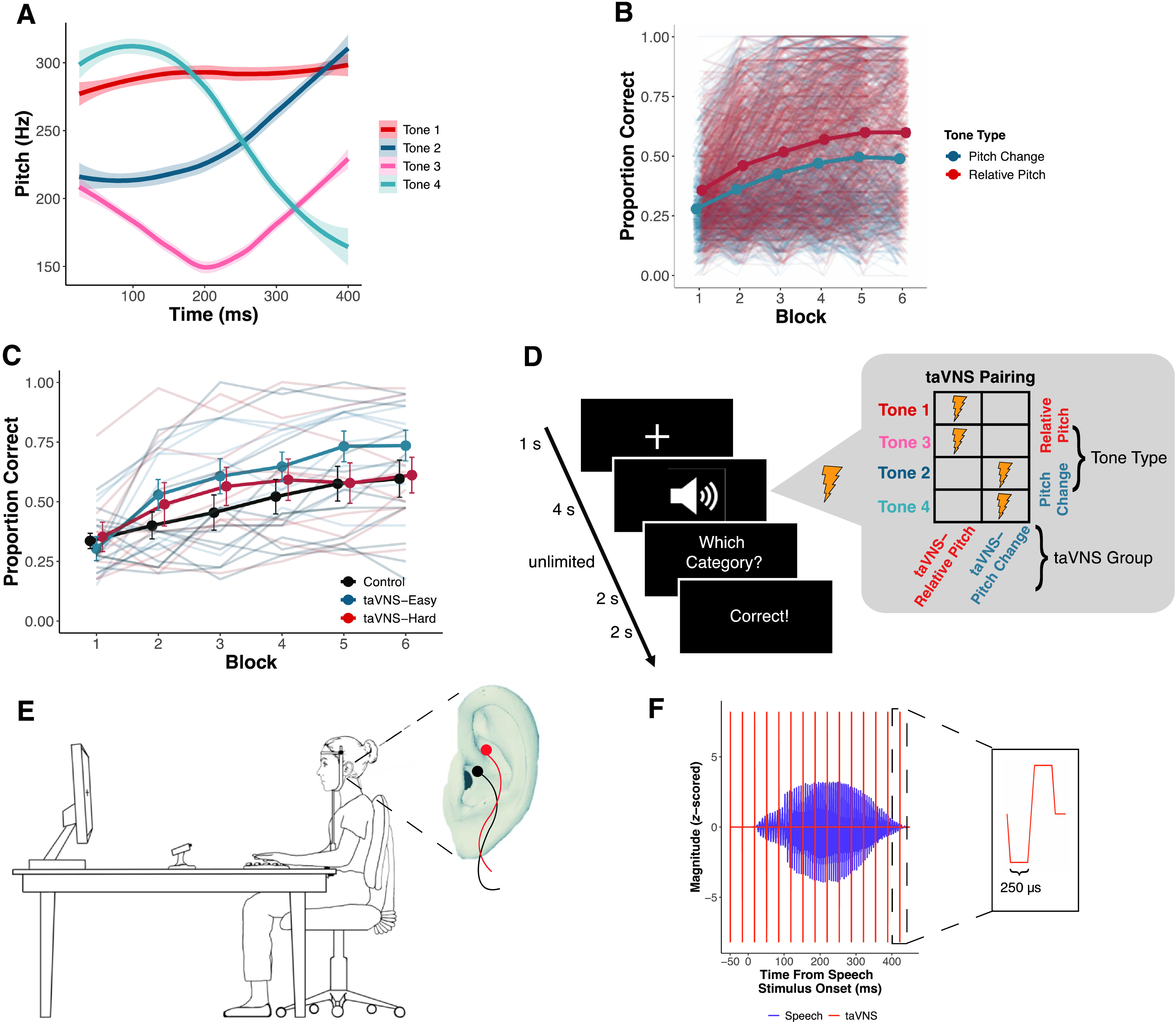
Methods. (A) Pitch contours (*M* and *SE*) of the four Mandarin tones spoken by two female speakers from the Mandarin tone category learning task. (B) Individual and mean learning performance of pitch change (Tone 2, Tone 4) and relative pitch (Tone 1, Tone 3) tones in an aggregate data set of 678 listeners across eight published studies reported by Llanos et al. (2020). Listeners are overall better at categorizing relative pitch tones. (C) Individual and mean learning performance by Llanos et al. (2020). The taVNS–easy and taVNS–hard groups correspond to taVNS–relative pitch and taVNS–pitch change in this study, respectively. (D) Mandarin tone category learning task trial structure. Stimulation pairing with tone categories is shown for each taVNS group. (E) Schematic of participant completing Mandarin tone category learning task with pupillometry. The placement of taVNS electrodes on cymba concha and cymba cavum is shown in a close up of the left ear. (F) taVNS–speech stimulus alignment on an example trial. taVNS consisted of trains of biphasic square wave pulses (inset). taVNS = transcutaneous auricular vagus nerve stimulation.

In the study of Llanos et al. (2020), subthreshold taVNS was paired with either relative pitch tones (Tone 1, Tone 3) or pitch change tones (Tone 2, Tone 4). These taVNS tone pairings were chosen after analyzing Mandarin tone category learning performance in more than 675 native English listeners, which found that performance on tones that are primarily differentiated on the basis of pitch change was significantly poorer than performance on relative pitch tones (see Figure 1B; Llanos et al., 2020). Pairing taVNS with the easier-to-learn relative pitch tones led to significantly better learning performance compared to those who received taVNS paired with the harder-to-learn pitch change tones or a control group who received no taVNS during the category learning task (see Figure 1C). These findings suggest that taVNS selectively enhanced recognition of nonnative speech sounds during learning. However, this study was only able to speculate as to the underlying mechanisms by which taVNS affected behavioral performance.

One of the primary mechanisms of action by which VNS, in general, and taVNS, in particular, are hypothesized to affect brain activity and behavior is via noradrenergic modulation of arousal (Schuerman et al., 2022; Vonck & Larsen, 2018). Increases in arousal have been found to affect neural plasticity, biasing memory consolidation toward perceptual objects with greater perceptual salience or top-down relevance (Mather & Sutherland, 2011). Though numerous neurobiological pathways contribute to arousal, the locus coeruleus–norepinephrine (LC-NE) system is one of the key systems that is strongly associated with arousal (Aston-Jones & Cohen, 2005; McGinley et al., 2015). Activation of the LC releases NE into numerous cortical structures (Aston-Jones & Cohen, 2005), which can modulate processing of sensory information (Berridge & Waterhouse, 2003). The LC-NE system operates via two modes of action, phasic and tonic modes, each of which can influence brain activity and behavior. Phasic LC activity is characterized by task-oriented behaviors to optimize performance, while tonic LC activity is associated with task disengagement and exploration for alternative behaviors. During phasic mode, neuronal firing rates are low at baseline and demonstrate a strong NE response to task-relevant stimuli, which is associated with higher task performance (Aston-Jones & Cohen, 2005). In contrast, LC neurons during tonic mode show elevated activation during baseline activity, which is associated with a global increase in NE and poorer task performance (Aston-Jones & Cohen, 2005). An individual's arousal state is driven by the balance between these phasic and tonic modes and has been shown to be affected by VNS (Hulsey et al., 2017) and taVNS (Garcia et al., 2017, 2021; Sclocco et al., 2019; Yakunina et al., 2017). Therefore, combining taVNS with a physiological measure of arousal and noradrenergic activity would allow for better examination of the mechanism of action for taVNS behavioral effects.

Prior research has implicated several potential biomarkers of effective VNS on arousal and noradrenergic activity. These include electrophysiological increases in P300 amplitude of event-related potentials, salivary responses, and pupillary dilation (Burger, D'Agostini, et al., 2020; Farmer et al., 2021; Nonis et al., 2017). However, the modulatory effects of VNS on these markers have been inconsistent. Regarding the P300, some studies have documented increases in the P300 associated with VNS (Lewine et al., 2019; Rufener et al., 2018), while others observed no changes in P300 amplitude (Fischer et al., 2018; Warren et al., 2019). Pupillary responses are a well-documented indicator of noradrenergic and LC-NE system activity in animals (Aston-Jones & Cohen, 2005; Joshi et al., 2016; Liu et al., 2017; Reimer et al., 2016). VNS in animals has also been shown to modulate LC neuronal firing (Bianca & Komisaruk, 2007; Garcia et al., 2017, 2021; Hulsey et al., 2017; Mridha et al., 2021; Sclocco et al., 2019; Yakunina et al., 2017).

Evidence for VNS modulation of pupillary responses in humans, however, is less clear. Some studies measuring phasic pupillary responses have observed changes in baseline pupil size associated with taVNS (Sharon et al., 2021; Urbin et al., 2021), while others observed no changes (Burger, Van der Does, et al., 2020). Additionally, the results of studies investigating effects on task-evoked pupillary responses have been inconsistent for both implanted VNS and taVNS. For example, Jodoin et al. (2015) found that pupillary responses were modulated by implanted VNS, but Schevernels et al. (2016) observed no relationship between pupillary responses and implanted VNS. In taVNS, pupillary responses showed no effect compared to sham stimulation during an active task (Burger, Van der Does, et al., 2020; Keute et al., 2019; Warren et al., 2019). Pandža et al. (2020) observed an effect of taVNS on the pupillary response in humans during a Mandarin tone category learning task. Individuals who received taVNS before each trial in the learning task demonstrated a sharper drop off in the pupillary response across training days than individuals in a sham taVNS condition (Pandža et al., 2020). Given the conflicting results across studies, a consistently reliable marker of taVNS efficacy in humans has yet to be established.

This study aimed to evaluate pupillary responses to taVNS during a nonnative speech category learning task. We adapted the Mandarin tone category learning task from Llanos et al. (2020), in which taVNS facilitated behavioral learning performance when paired with easier-to-learn relative pitch tones. We combined subthreshold taVNS, pupillometry, and Mandarin tone category learning into a single experimental design, with the goal of examining how taVNS behavioral effects may be reflected in the pupillary response. Deviating from the design in the Llanos et al. study, we delayed the timing of the Mandarin tone training task trial events to prioritize pupillometry data collection and modified taVNS parameters to match those that were found to elicit the most consistent changes in widespread neural activity (cf. Schuerman et al., 2021; see Figures 3B–3C), putatively driven by vagal activation (e.g., Cao et al., 2017).

In a preregistered protocol (https://osf.io/zw9m7; Schuerman et al., 2019), we trained native English-speaking adults to categorize Mandarin Chinese tones. In contrast to prior studies that measured pupillometry in blocks where VNS was turned on compared to blocks with VNS turned off (Jodoin et al., 2015; Keute et al., 2019; Schevernels et al., 2016), here the presence of VNS varied on a trial-by-trial basis, depending on which Mandarin tone was presented on a given trial. Following the same taVNS–tone category pairing as in the study of Llanos et al. (2020), in a double-blinded design participants were randomly divided into two taVNS groups that differed according to which tones were paired with taVNS: (a) taVNS paired with relative pitch tones (taVNS–relative pitch; Tone 1 and Tone 3) and (b) taVNS paired with pitch change tones (taVNS–pitch change; Tone 2 and Tone 4). We first hypothesized that our behavioral results would accord with the Llanos et al. study, such that the taVNS–relative pitch group would exhibit greater overall learning performance compared to the taVNS–pitch change group. Because only half of the Mandarin tones were paired with taVNS, we also hypothesized that pairing taVNS with auditory stimuli would increase the magnitude of the tone-evoked pupillary responses compared to auditory stimuli that were not paired with taVNS.

In addition to the preregistered analyses, we also explored the extent to which taVNS amplitude impacted learning performance, response times, and pupillary responses. Prior studies in animals (Borland et al., 2016; Loerwald et al., 2018; Pruitt et al., 2021) and humans (Clark et al., 1999) have demonstrated that stimulation amplitude is a key parameter for modulation of neuroplasticity. Clark et al. (1999) found that VNS at 0.5 mA elicited greater average word recognition scores compared to stimulation between 0.75 and 1.5 mA or no VNS. We hypothesized that lower taVNS amplitudes may lead to better tone category learning performance than higher taVNS amplitudes. A similar Mandarin tone category learning study observed a reduction in response times across training days but no differences in response times within a single session as a function of taVNS (Pandža et al., 2020). Here, we also examined the extent to which taVNS amplitudes impacted response times across the training task. Slower response times are associated with poorer accuracy on decision-making tasks, but faster response times can also come at the cost of poorer accuracy (Binder et al., 2004). Therefore, we hypothesized that response times would reduce across the training task, which would indicate higher accuracy and better learning performance. We also hypothesized that the reduction in response times would differ as a function of taVNS amplitudes. Given that we hypothesized that lower taVNS amplitudes would lead to greater accuracy across the training task, we hypothesized that those with lower taVNS amplitudes would have a larger reduction in response times across training.

With regard to pupillary responses, we also conducted growth curve analyses (GCAs) to understand the extent to which different components of the pupillary response may be affected by taVNS. Our preregistered analyses were designed to use mean and maximum pupillary measures, which may miss subtle aspects of the influence of taVNS on pupillary responses. However, the pupillary response unfolds over time and often does not follow a linear trajectory. Therefore, analyzing the mean and maximum change in pupillary dilation alone does not accurately capture the functional form of the pupillary response. GCA models time-series data, making it an appropriate analysis for modeling changes in the pupillary response over time. We hypothesized that trials paired with taVNS would have larger GCA parameters compared to trials without taVNS and that higher taVNS amplitudes would show greater effects on the GCA parameters compared to lower taVNS amplitudes.

## Method

### Participants

Forty-two participants between the ages of 18 and 33 years (*M* = 22.86, *SD* = 4.12) were recruited for this study from the greater Pittsburgh community. All participants were native English speakers and had hearing thresholds of < 25 dB for frequencies 250–8000 Hz in octave steps. Participants completed a language history questionnaire to ensure no prior exposure to tonal languages (language courses, immersion experiences, etc.). At the beginning of the experiment, participants were randomly assigned to one of two participant groups in a double-blinded design: taVNS–relative pitch (*n* = 21) and taVNS–pitch change (*n* = 21). Four participants were excluded from analyses after completion of the experiment: One participant disclosed experience with Mandarin after completing all portions of the experiment, which had not been reported on their language history questionnaire; the taVNS device shut off midexperiment for one participant; and two participants had less than 15 (out of 200) usable trials for pupillometry (postprocessing). This resulted in final group totals of 18 participants in the taVNS–relative pitch group and 20 participants in the taVNS–pitch change group. Participant demographics are displayed in Table 1.

**Table 1.** Participant demographics.

Variable	All	taVNS–relative pitch	taVNS–pitch change
*n* (female)	38 (19)	18 (7)	20 (12)
Mean age in years (*SD*)	22.737 (4.105)	22.667 (4.116)	22.800 (4.200)
Mean music experience in years (*SD*)	2.316 (2.569)	1.833 (2.229)	2.750 (2.826)
Mean taVNS amplitude (range)	0.692 (0.1–1.5)	0.717 (0.1–1.4)	0.670 (0.1–1.5)

*Note.* taVNS = transcutaneous auricular vagus nerve stimulation.

Prior work suggests musical experience influences speech processing (Bidelman et al., 2011; Schön et al., 2004; Wong et al., 2007). As such, participants completed a music history questionnaire prior to the experiment. Musical experience between taVNS groups did not significantly differ, *t*(35.43) = −1.115, *p* = .272. Additionally, the number of years of music experience in each group was less than the number of years shown to enhance learning in our nonnative speech category learning task (Smayda et al., 2015). Participants received monetary compensation for their participation. This research protocol was approved by the institutional review board at the University of Pittsburgh.

### Stimuli

Participants learned to categorize the four Mandarin tones that vary along the relative pitch and pitch change acoustic dimensions. Four lexical tones were produced in five syllable contexts (/bu/, /di/, /lu/, /ma/, and /mi/) by four native Mandarin Chinese speakers (two females; Feng et al., 2018), resulting in a total of 80 unique speech stimuli (5 syllables × 4 talkers × 4 tones). The Mandarin tone stimuli were root-mean-square amplitude (70 dB) and duration (442 ms) normalized using Praat (Boersma & Weenink, 2005).

### Procedure

#### Category Learning Task

Monocular left eye pupil size data were monitored and recorded at 1000 Hz using an Eyelink 1000 Plus Desktop Mount with a chin and forehead rest for stabilization. Luminance of the visual field was controlled with consistent ambient lighting across all participants. Nine-point eye tracker calibration was performed prior to the start of the Mandarin tone category learning task.

Participants completed two sections of the Mandarin tone category learning task while pupillometry was recorded. During the training section, participants learned to categorize the Mandarin tones across five blocks. Half of the Mandarin tone stimuli (two talkers; one female) were presented once per block for a total of 40 trials per block and 200 trials across training. Consistent with prior research, time delays were included after each trial event to capture the entirety of the pupil response, which unfolds over several seconds (Koelewijn et al., 2018; McHaney et al., 2021; Winn et al., 2018; Zekveld et al., 2013). Each trial in the training task began with a fixation cross. Participants were required to fixate on the cross in the center of the screen for a minimum of 1,000 ms to begin each trial. The mandatory fixation criteria helped to control for the effects of saccades, which affect pupillary measurements, and to minimize pupil foreshortening errors (Hayes & Petrov, 2016). The speech stimulus for that trial was presented upon meeting the fixation criterion. There was a 4-s delay between the onset of the speech stimulus and the category response prompt, which replaced the fixation cross on the screen with the words “Which category?” Participants were required to provide a keyboard response (1, 2, 3, 4) to indicate the Mandarin tone category. There was a 2-s delay between participant response and the display of corrective feedback on the screen. This feedback consisted of the words “Correct” or “Wrong.” Feedback was displayed for 2 s. An example trial is depicted in Figure 1D. Participants received a self-timed break at the end of each block. Manual drift correction was performed by the experimenter before beginning each successive block to ensure high-quality tracking of the pupil throughout the training task. Participants completed a similar task during the second section of the task, referred to as the “generalization block”. The trial succession for the generalization block was the same as in the training task, except that corrective feedback was not provided and novel stimuli were used. Participants categorized the remaining Mandarin tone stimuli that were not presented in the training section (two speakers; one female; 40 trials total). The category learning task was created and presented using MATLAB 2018b (MathWorks, Inc.), and the speech stimuli were presented to the right ear via ER-3C insert earphones (Etymotic Research).

#### Peripheral Nerve Stimulation

During the category learning task, subthreshold stimulation was delivered on half of the trials, depending on the participant's taVNS group assignment (see Figure 1D). Participants in the taVNS–relative pitch group received stimulation on any trial where Tone 1 or Tone 3 was presented. These are the tones that primarily differ along the relative pitch dimension. Participants in the taVNS–pitch change group received stimulation on trials where Tone 2 or Tone 4 was presented, which primarily differ along the pitch change dimension.

To transcutaneously stimulate the vagus nerve, we targeted the cymba concha and cymba cavum of the left outer ear (see Figure 1E), which is innervated by the auricular branch of the vagus nerve (Badran, Brown, et al., 2018; Peuker & Filler, 2002). Stimulation was delivered to these sites at amplitudes below each participant's perceptual threshold. The perceptual threshold was identified using a 0.1-mA up/0.3-mA down staircase procedure prior to the start of the category learning task. The threshold was calculated as the average stimulation amplitude after eight reversals (Llanos et al., 2020). During the training section of the Mandarin tone category learning task, stimulation was delivered with a pulse amplitude of 0.2 mA below the participant's perceptual threshold (Llanos et al., 2020; Schuerman et al., 2021). Maximum pulse amplitude was limited to 3.0 mA due to safety restrictions.

To apply stimulation, the participant's left ear was first cleaned with alcohol and an abrasive gel to remove excess oil from the skin. Silicon putty was then molded to the shape of their ear. Two Ag-AgCl disc electrodes (4-mm diameter) were embedded into the putty at the cymba concha and cymba cavum locations. A salt-free conductive gel was applied to the electrode, and the putty mold was reinserted into the left ear. Stimulation was generated with a BIOPAC STMISOLA Constant Current Isolated Linear Stimulator. Stimulation parameters consisted of 15 biphasic square wave pulses with a 250-μs pulse width, delivered at a rate of 30 Hz. The biphasic waveforms were generated using MATLAB 2018b (MathWorks, Inc.) and transmitted to the stimulator via a National Instruments USB-6211 DAQ card. The pulse train began approximately 50 ms prior to the onset of the speech stimulus and continued for 500 ms through the entire presentation of the speech stimulus. The speech stimulus and pulse train alignment are depicted in Figure 1F. Average stimulation amplitude did not significantly differ between taVNS groups, *t*(35.74) = 0.331, *p* = .742.

#### Discrimination Task

Participants in the taVNS groups also completed a tone discrimination task before and after the category learning task. During this task, participants performed speeded discrimination judgments of Mandarin tone pairs and were required to indicate via keyboard press whether the tone pairs were “same” or “different” (AX discrimination) as quickly and as accuracy as possible. Tone contours based on the fundamental frequency of the talker were modeled for all four Mandarin tones and were superimposed on the vowel /a/ using the pitch-synchronous overlap and add method implemented in Praat (Boersma & Weenink, 2005). Stimuli had a duration of 250 ms. During the discrimination task, pupillometry was not recorded, and participants did not receive stimulation.

Participants first completed a five-trial practice phase to gain familiarity with the discrimination task. Each trial consisted of a pair of stimuli and a 500-ms interstimulus interval. Participants then completed the discrimination task, which comprised a total of 240 trials. The “same” and “different” trials had equal probability of occurrence (*p* = .500). All trials were randomized within each block. Participants had unlimited time to respond after each trial.

### Analyses

#### Preregistered Analyses

This study was preregistered at https://osf.io/zw9m7 (Schuerman et al., 2019). The analyses listed below were included in the preregistration.

##### Tone category learning performance per taVNS group

A binomial generalized linear mixed-effects model was fit to examine learning accuracy in the Mandarin tone category learning task using the lme4 package (Bates et al., 2015) in R (R Core Team, 2022). The outcome variable of the model was trial-by-trial accuracy (correct, incorrect) for each participant. The model included fixed effects of trial, tone category, and group, as well as all two- and three-way interactions between fixed effects. The model included a maximal random effect structure with random slopes of subject per trial, item per trial, and a random slope for the interaction between subject and tone category per trial: Accuracy ~ trial * tone category * group + (*trial* | *subject*) *+* (*trial* | *subject: tone category*) *+* (*trial* | *item*). This model provided a significantly lower Akaike information criterion (AIC) value than models with only a random intercept of trial per subject or intercepts of trial per subject and trial per item, χ^2^(3) = 139.920, *p* < .001 (AIC_(trial|subject)_ = 8,847.543; AIC_(trial|subject) + (trial|wav)_ = 8,670.264; AIC_final_ = 8,536.341). The residual plot of the final model was analyzed to confirm linearity and homoscedasticity. The residuals were also visually inspected via histograms and Q–Q plots to confirm normality. The multcomp package (Hothorn et al., 2008) in R was used to examine all pairwise comparisons between tone categories and groups. Bonferroni-adjusted *p* values are reported.

##### Pupillometry

Consistent with prior research, pupillometry data from the training portion of the Mandarin tone category learning task were preprocessed to remove noise from the analysis (McHaney et al., 2021). Pupillometry data were down-sampled to 50 Hz, and trials with more than 15% of the samples detected as blinks were removed (*n* = 1,134 out of 7,600 trials). Missing samples due to blinks were linearly interpolated to 140 ms before and after the detected blink. Additional blinks were identified and linearly interpolated based on the first derivative of the blink threshold. Pupil responses were baseline normalized on a trial-by-trial basis using the average 500 ms prior to the onset of the taVNS. If taVNS was not present on a given trial, pupil responses were baseline normalized using the −560 to −60 ms period prior to the speech stimulus onset to mirror the same baseline period used to normalize taVNS trials. The outcome variable reported is the proportion change in pupil size relative to baseline in arbitrary units.

Separate linear mixed-effects models were estimated to examine mean and max pupillary dilation time-locked to the speech stimuli during the Mandarin tone category learning task. The lme4 (Bates et al., 2015) and lmerTest (Kuznetsova et al., 2017) packages in R (R Core Team, 2022) were used to fit the models and to calculate *p* values. The generalization block was excluded from pupillary analyses. Pupillary data were averaged for subject and item. Fixed effects included main effects of tone category, taVNS group, and the interaction between tone and taVNS group. A model containing a random intercept of subject and a random slope of taVNS group per item provided a singular fit; thus, the final model included only a random intercept of subject: Pupil ~ tone category * group + (1 | *subject*). The residual plot of this model was analyzed to confirm linearity and homoscedasticity. The residuals were also visually inspected via histograms and Q–Q plots to confirm normality. Multiple comparisons were conducted using the multcomp package (Hothorn et al., 2008) in R to examine all pairwise comparisons between tone categories and taVNS groups. We report Bonferroni-adjusted *p* values.

##### Cluster-based permutation analysis

To determine whether tone category or group-level differences existed at any point in the time course of the response, we employed cluster-based permutation tests (Maris & Oostenveld, 2007) adapted to the design of the experiment. Linear mixed-effects models were fit with the same fixed effect structure utilized for the mean and max pupil response models. Due to the large number of models, the structure of random effects consisted of random intercepts for participant and item. For each model, the dependent variable was the pupillary response at each time point after onset of auditory stimulation presentation. As with the mean and max pupil analyses, we excluded the generalization block from analysis. *F* values for each fixed effect term (tone category, taVNS group, and the two-way interaction) were extracted. Degrees of freedom and *p* values were estimated using the Satterthwaite approximation. Contiguous *F* values corresponding to *p* values < .05 were grouped into temporal clusters, and the *F* values for each cluster were summed. These summed cluster values constituted the test statistic to compare against corresponding null distributions for each term. To generate the null distributions, we performed 1,000 random permutations of the data set. For each, we randomly shuffled tone category labels within each participant and group labels across participants in order to preserve the overall structure of the data set (i.e., the within-subjects variable, tone category, was permuted within each participant, while the between-subjects group variable was permuted between participants). For each random permutation and each fixed-effect term, the maximum cluster sum value was extracted. These randomly generated cluster sums constituted a null distribution against which the true test statistic could be compared to compute a *p* value for significance.

#### Exploratory Analyses

Additional analyses were performed to examine differences between both taVNS groups, which were not originally included in the preregistration. These exploratory analyses are listed below.

##### Exploratory category learning analyses

While all participants received stimulation at the same amplitude relative to their perceptual threshold (threshold, 0.2 mA; see Peripheral Nerve Stimulation), absolute stimulation amplitude varied substantially between individuals (min = 0.1 mA, max = 1.5 mA, *M* = 0.71 mA, *SD* = 0.42 mA). Therefore, we fit a binomial generalized linear mixed-effects model to examine learning accuracy and a linear mixed-effects model to examine response times between stimulated and nonstimulated trials, regardless of taVNS group assignment, as a function of taVNS amplitude. For the binomial generalized linear mixed-effects model on accuracies, the outcome variable for this model was trial-by-trial accuracy during the training task. For the linear mixed-effects model, the outcome variable was trial-by-trial response times. Fixed effects included trial, stimulation (reference = nonstimulated trials), taVNS amplitude, and all two- and three-way interactions between trial, stimulation, and taVNS amplitude.

The maximal random effect structure that promoted convergence and a nonsingular fit for the model of accuracies included a random intercept of subject, a random slope of trial per subject that removed the correlation between trial and subject (Barr et al., 2013), a random slope of trial per item, and a random slope of trial per the interaction of subject and stimulation to properly group subject and stimulation: Accuracy ~ trial * stimulation * taVNS amplitude + (1 | *subject*) *+* (0 + *trial* | *subject*) *+* (*trial* | *subject: stimulation*) + (*trial* | *item*)*.*

The maximal random effect structure that promoted convergence and a nonsingular fit for the response time model included a random intercept of subject, a random slope of trial per subject that removed the correlation between trial and subject (Barr et al., 2013), a random intercept of item: Response Time ~ trial * stimulation * taVNS amplitude + (*trial* | *subject*) + (*trial* | *subject: stimulation*) + (*trial* | *item*). The residual plot of each model was analyzed to confirm linearity and homoscedasticity. The residuals were also visually inspected via histograms and Q–Q plots to confirm normality.

##### GCA

Pupillary responses from −60 to 3,980 ms time-locked to speech stimulus onset were analyzed using GCA (Mirman, 2014). GCA uses orthogonal polynomials to capture distinct functional forms of the pupillary response. A GCA was fit to model the interactions between first-, second-, and third-order orthogonal polynomials. The third-order model provides three parameters to map the complexity of the pupillary response. The intercept reflects the overall change in the pupillary response over the entire time window and can be interpreted as the average change in the pupillary response from start to finish (Mirman, 2014). The linear (ot1) term represents the slope, or rate of dilation (if positive) and rate of constriction (if negative), of the pupillary response over time (Kuchinsky et al., 2014; Morett et al., 2020). The quadratic (ot2) term reflects the curvature of the pupillary response. Based on the trajectory of the pupillary response, the quadratic term should be negative. Here, a larger, negative quadratic term can be interpreted as more parabolic, while a quadratic term closer to zero reflects a more linear shape (Kuchinsky et al., 2014). The cubic (ot3) term represents the extent to which two inflection points occur in the pupillary response. For all terms, the absolute value of the term reflects the strength of the response, while the sign (i.e., positive or negative) reflects the direction of the response (Kuchinsky et al., 2014). GCA was conducted using the lme4 package (Bates et al., 2015) with log-likelihood maximization using the BOBYQA optimizer to promote convergence, and *p*-values were estimated using the lmerTest package (Kuznetsova et al., 2017).

We estimated a GCA to examine the effect of taVNS amplitudes on the pupillary responses on stimulated and nonstimulated trials, regardless of taVNS group assignment. The maximal final model that promoted convergence and a nonsingular fit included fixed effects of stimulation (reference = nonstimulated trials), taVNS amplitude, and the interaction of stimulation and taVNS amplitude on all time terms. The model included random slopes of subject on each time term, a random intercept for the interaction of subject and stimulation, and a random slope for the interaction of subject and stimulation on each time term that removed the correlation between time terms and interaction of subject and stimulation (Barr et al., 2013): *Pupil ~* (*ot*1 + *ot*2 + *ot*3) * Stimulation * taVNS Amplitude + (*ot*1 + *ot*2 + *ot*3 | *subject*) + (1 | *subject*: *Stimulation*) + (0 + *ot*1 + *ot*2 + *ot*3 | *subject*: *Stimulation*). This random effect structure provided a better model fit than when including only a random slope of subject on each time term, χ^2^(7) = 6845.8, *p* < .001 (AIC_M1_ = −79,142; AIC_M2_ = −72,310).

##### Discrimination analysis

Linear mixed-effects models were fit to examine accuracies and response times from the speech discrimination task at pre- and posttraining by participant group. For each model, the fixed effects were taVNS group (reference = taVNS–relative pitch), testing time (reference = pretraining), and the interaction between taVNS group and testing time. A random intercept of subject was included in each model.

##### Individual differences scaling

Multidimensional scaling analyses were conducted on the reaction time data obtained from the speech discrimination task. The assumption was that the perceptual distance between two auditory objects can be determined from the time taken to discriminate between the two sounds (Nosofsky, 1992), wherein larger reaction times would suggest stimuli are closer in perceptual space. Here, we utilized individual differences scaling (INDSCAL; Carroll & Chang, 1970). The INDSCAL output provides a group stimulus space, which represents the four Mandarin tones in Euclidian space. The distance between points in the group space is represented by a weighted Euclidean function. The weighting pattern for each participant contributing to the group space can then be analyzed to understand the importance of these different dimensions for each participant.

The input for the INDSCAL analysis consisted of 76 (38 participants at pretest and posttest) separate 4 tones × 4 tones symmetric data matrices. Each matrix contained distance estimates derived from the averaged inverse of reaction time (1/RT) for each paired comparison (T1 vs. T1, T1 vs. T2, T1 vs. T3, T1 vs. T4, T2 vs. T2, T2 vs. T3, T2 vs. T4, T3 vs. T3, T3 vs. T4, T4 vs. T4) in the task. The INDSCAL analysis was performed with two dimensions, which has shown to be the appropriate number of dimensions underlying the distances in the perceptual space of Mandarin tones in native English speakers (Chandrasekaran et al., 2007, 2010). A two-way analysis of variance (ANOVA) on the average participant weights per group per assessment time (pretraining vs. posttraining) was conducted for each perceptual dimension. Bonferroni-adjusted *p* values are reported to correct for multiple comparisons.

## Results

**Figure 2.**
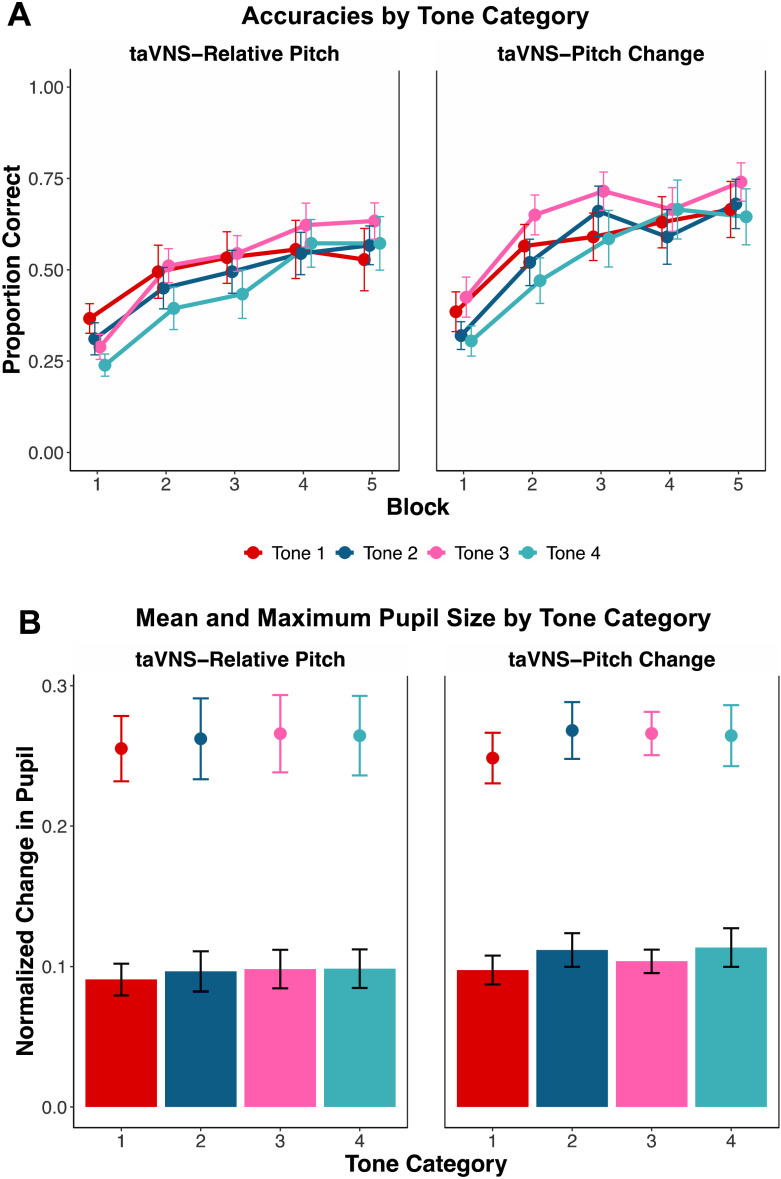
Behavior and pupillometry per tone category. (A) Categorization accuracy and standard error of the mean for each of the four Mandarin tone categories across five blocks of training, split by taVNS group. Categorization accuracy is defined as proportion of correct trials within each block. (B) Change in pupil size as a function of tone category for each taVNS group. The mean (bar height) and maximum (point) pupil responses are shown with error bars denoting standard error of the mean. taVNS = transcutaneous auricular vagus nerve stimulation.

First, we examined accuracy on the four tone categories in each group during the five blocks of training (see Figure 2A). The binomial generalized linear mixed-effects model revealed a significant effect of trial (β = .365, *z* = 2.147, *p* = .032), which indicates that participants had greater accuracy at the end of training than at the beginning, suggesting that learning did occur. However, we did not observe a significant effect of taVNS group (β = .416, *z* = 1.008, *p* = .313) or a significant interaction of trial and taVNS group (β = .257, *z* = 1.139, *p* = .255), indicating that taVNS groups did not differ in their overall accuracy nor their trial-by-trial e_k; increase in accuracy. Multiple pairwise comparisons also did not reveal any significant effects of tone category; interactions of trial and tone category; nor the interactions of trial, taVNS group, and tone category (*p*s > .05). These results suggest that taVNS did not have any tone-specific or group enhancements to learning accuracy.

Next, we assessed whether mean and max pupillary responses time-locked to the onset of the speech stimulus differed between tone categories, taVNS groups, or between taVNS groups for specific tone categories (see Figure 2B). When examining mean pupillary responses, we did not observe a significant effect of tone category (*p*s > .05), taVNS group (β = .007, *t* = 0.397, *p* = .693), or a significant interaction between tone category and taVNS group (*p*s > .05). For the maximum pupillary response, we also did not observe any significant effects of tone category (*p*s > .05), taVNS group (β = −.007, *z* = −0.028, *p* = .836), or interactions between tone category and taVNS group (*p*s > .05). Taken together, these results suggest that taVNS did not have any tone-specific effects on the average pupillary response and the peak dilation size between taVNS groups.

The cluster-based permutation analyses on the pupillary response revealed the presence of one nonsignificant cluster for the main effect of tone (*p* = .062), extending from 2.05 s after sound onset to 3.25 s after sound onset. Averaging over this window, the largest pupillary response was found for Tone 2 (*M =* 11.77%), followed by Tone 4 (*M =* 11.69%), Tone 3 (*M =* 11.36%), and Tone 1 (*M =* 9.83%). No significant clusters were found for the main effect of taVNS group or the interaction between taVNS group and tone category.

### Exploratory Analyses

#### Effect of taVNS Amplitude on Category Learning Performance and Response Times

**Figure 3.**
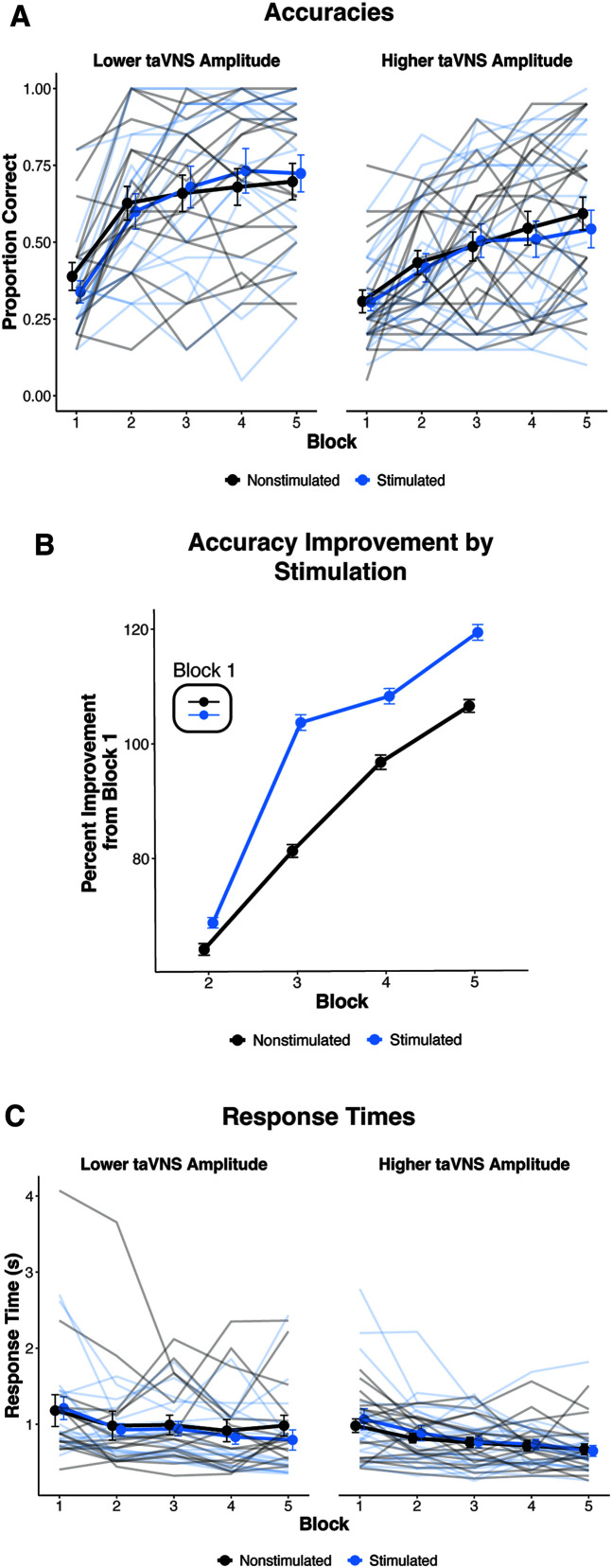
Exploratory results: category learning performance and response times. (A) Average categorization accuracy as a function of stimulation (i.e., trials paired with taVNS or no taVNS) for individuals who received taVNS at lower amplitudes versus higher amplitudes. (B) Average percent improvement in learning performance relative to Block 1 accuracy (inset) to illustrate rate of learning between stimulated and nonstimulated trials. (C) Average response times as a function of stimulation did not differ across training for those who received taVNS at lower amplitudes versus higher amplitudes. Participants were split into lower and higher taVNS amplitude groups based on a median split (*Mdn* = 0.6 mA) for visualization purposes only. Error bars represent standard error of the mean. Average categorization accuracies for stimulated and nonstimulated trials are denoted by the darker lines and points. The lighter lines denote individual differences in category learning performance. taVNS = transcutaneous auricular vagus nerve stimulation.

In addition to the preregistered analyses, we examined the effect of stimulation (i.e., presence or absence of taVNS on a given trial) and taVNS amplitude on categorization accuracy and response times across training, regardless of taVNS group assignment (see Figure 3).

The binomial generalized linear mixed-effects model of accuracies revealed a significant effect of taVNS amplitude (β = −.810, *z* = −2.124, *p* = .034), indicating that, on average, individuals who received taVNS at higher amplitudes had fewer correct responses during training than those who received taVNS at lower amplitudes. We did not observe a significant interaction of trial and taVNS amplitude, which suggests that the rate of learning did not differ based on taVNS amplitude.

We observed a significant, positive interaction of trial and stimulation (β = .340, *z* = 2.079, *p* = .038). This significant interaction suggests that stimulated trials had a larger trial-by-trial increase in accuracy across the task compared to nonstimulated trials. Additionally, the three-way interaction of trial, taVNS amplitude, and stimulation was significant (β = −.401, *z* = −2.029, *p* = .042; see Figure 3B). This significant interaction indicates that the difference in the rate of learning between stimulated and nonstimulated trials gets significantly smaller as taVNS amplitudes increase. Independent of the effect of trial number, we did not observe a significant main effect of stimulation (β = .089, *z* = 0.490, *p* = .624) or interaction between stimulation and taVNS amplitude (β = −.133, *z* = −0.604, *p* = .546). This indicates that overall accuracy did not differ between stimulated and nonstimulated trials or on the basis of taVNS amplitude. Collectively, these behavioral results demonstrate a general effect of taVNS on learning rate, which was stronger for individuals who received lower taVNS amplitudes.

The linear mixed-effects model of response times revealed no significant effect of trial (β = −.105, *t* = −1.607, *p* = .114). The lack of a significant effect of trial indicates that response times did not become faster or slower as training progressed. There were also no significant effects of amplitude (β = −.156, *t* = −1.041, *p* = .304); stimulation (β = −.098, *t* = −1.509, *p* = .140); nor interactions of trial and amplitude (β = .027, *t* = 0.332, *p* = .741), trial and stimulation (β = −.048, *t* = −0.838, *p* = .408), amplitude and stimulation (β = .122, *t* = 1.514, *p* = .139), and trial, amplitude, and stimulation (β = −.012, *t* = −0.168, *p* = .867). Collectively, this model indicates that the presence of taVNS on a given trial and taVNS amplitude did not modulate response times.

#### Pupillary Responses Not Modulated by taVNS

**Figure 4.**
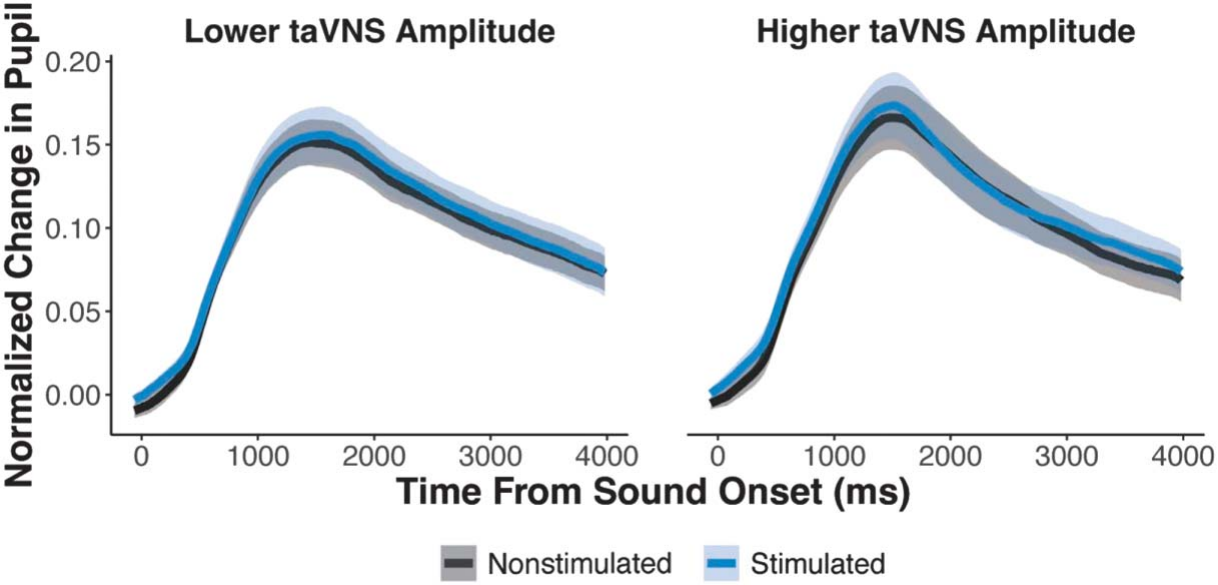
Pupillary responses to stimulated versus nonstimulated trials. Baseline normalized pupillary responses to stimulated versus nonstimulated trials for individuals that received lower versus higher taVNS amplitudes. For visualization purposes only, participants were split into lower and higher taVNS amplitude groups based on a median split (*Mdn* = 0.6 mA). Shaded regions represent standard error of the mean. taVNS = transcutaneous auricular vagus nerve stimulation.

For pupillary responses, we first used GCA to examine the pupil response on stimulated versus nonstimulated trials, regardless of taVNS group assignment (see Figure 4 and Table 2). Overall, the average pupillary responses between stimulated and nonstimulated trials did not significantly differ (β = .009, *p* = .241). Additionally, there were no significant effects of stimulation on the linear, quadratic, or cubic terms (*p*s > .05). Thus, the presence of taVNS on a given trial did not modulate the pupillary response.

**Table 2.** Fixed-effect estimates for model of pupillary responses from −60 to 3,980 ms time-locked to the speech stimulus onset to examine the effect of stimulation and transcutaneous auricular vagus nerve stimulation (taVNS) amplitude (observations = 15,428).

Fixed effect	Estimate	*SE*	95% CI	*t*	*p*
Intercept	0.100	0.015	[0.070, 0.131]	6.539	< .001
ot1	0.188	0.083	[0.025, 0.351]	2.261	.024
ot2	−0.508	0.095	[−0.695, −0.322]	−5.350	< .001
ot3	0.263	0.046	[0.173, 0.354]	5.706	< .001
Stimulation (stimulated vs. nonstimulated trials)	0.009	0.008	[−0.006, 0.024]	1.192	.233
ot1 × Stimulation	0.030	0.047	[−0.063, 0.123]	0.624	.533
ot2 × Stimulation	−0.008	0.031	[−0.068, 0.051]	−0.277	.782
ot3 × Stimulation	−0.033	0.023	[−0.078, 0.011]	−1.457	.145
taVNS amplitude	−0.004	0.019	[−0.041, 0.033]	−0.192	.847
ot1 × taVNS Amplitude	−0.021	0.103	[−0.222, 0.181]	−0.201	.841
ot2 × taVNS Amplitude	−0.083	0.117	[−0.312, 0.147]	−0.707	.480
ot3 × taVNS Amplitude	−0.044	0.057	[−0.156, 0.067]	−0.776	.438
Stimulation × taVNS Amplitude	−0.006	0.009	[−0.025, 0.012]	−0.691	.490
ot1 × Stimulation × taVNS Amplitude	−0.063	0.058	[−0.178, 0.052]	−1.072	.284
ot2 × Stimulation × taVNS Amplitude	0.039	0.038	[−0.035, 0.113]	1.029	.304
ot3 × Stimulation × taVNS Amplitude	0.051	0.028	[0.004, 0.106]	1.821	.069

*Note.* Growth curve model: lmer(Pupil ~ (ot1 + ot2 + ot3) * taVNS amplitude * Stimulation + (ot1 + ot2 + ot3 | Subject) + (1 | Subject:Stimulation) + (0 + ot1 + ot2 + ot3 | Subject:Stimulation), control = lmerControl(optimizer = “bobyqa”)). CI = confidence interval; ot1 = linear; ot2 = quadratic; ot3 = cubic.

Next, we examined the extent to which pupillary responses were modulated by the amplitude of taVNS. The GCA revealed no significant main effect of taVNS amplitude (β = −.004, *p* = .848) or significant interaction between amplitude and the linear, quadratic, or cubic terms (*p*s > .05). Additionally, the interactions of stimulation and taVNS amplitude were not significant for the intercept, linear, quadratic, or cubic terms (*p*s > .05). Collectively, the GCA results suggest that neither the presence of taVNS on a given trial nor the amplitude of stimulation modulated pupillary responses in our task.

#### Cue Weighting Does Not Differ Between Pre- and Posttraining

**Figure 5.**
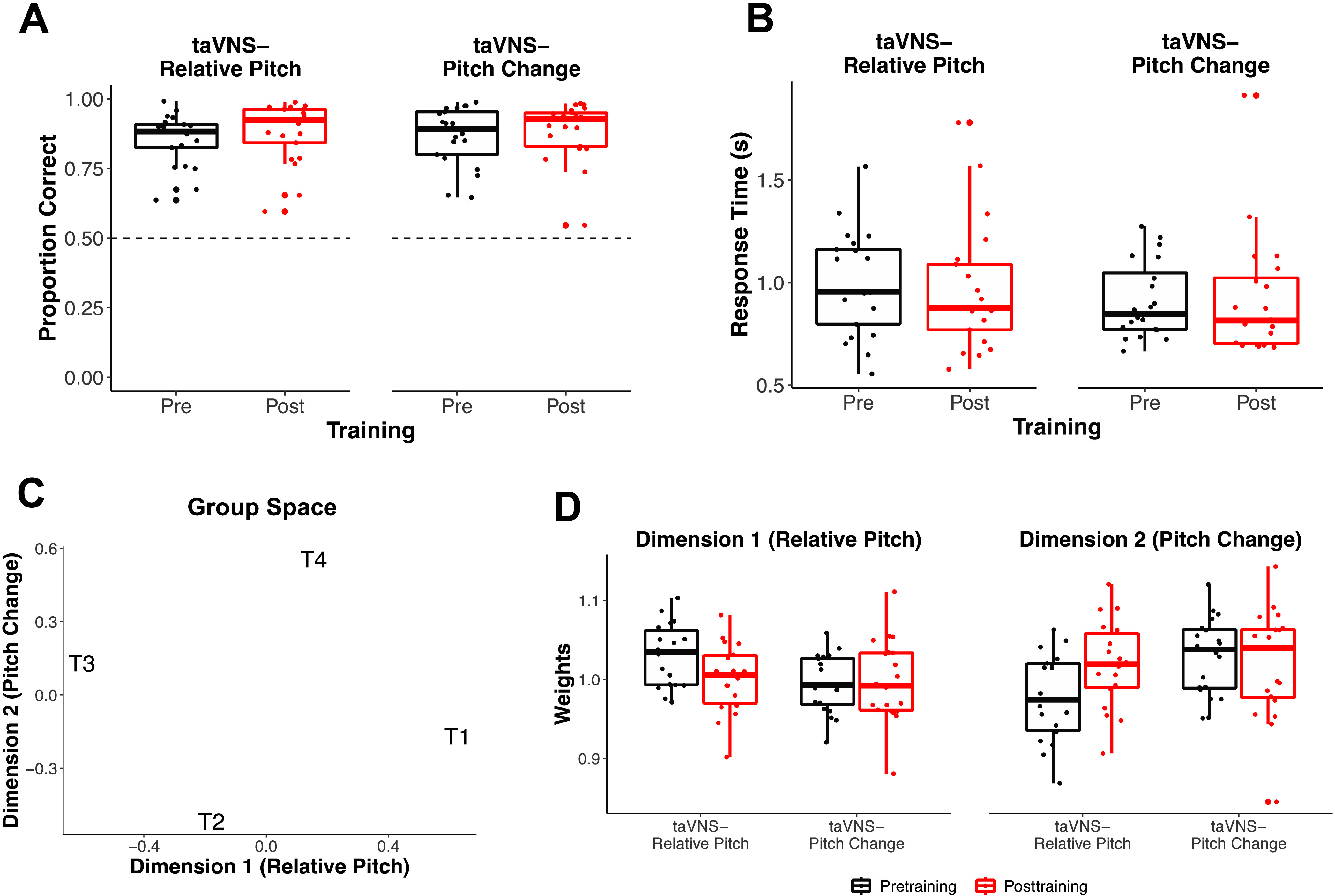
Speech discrimination task analysis. (A) Average accuracies for each taVNS group on the speech discrimination task did not differ at pre- or posttraining. The dashed line reflects chance. Individual participant accuracies are denoted by points. (B) Average response times did not differ at pre- or posttraining across taVNS groups. (C) Group stimulus space obtained from the INDSCAL analyses on the inverse of response times. Tone 1 (T1) and Tone 3 (T1) are located the furthest apart along Dimension 1, interpretatively labeled as relative pitch. Tone 2 (T2) and Tone 4 (T4) are the farthest apart along Dimension 2 (pitch change). The perceptual dimensions from the INDSCAL are consistent with the dominant acoustics of the training task stimuli set. (D) Average perceptual weights of Dimension 1 and Dimension 2 for each taVNS group did not differ at pre- or posttraining. taVNS = transcutaneous auricular vagus nerve stimulation; INDSCAL = individual differences scaling.

First, accuracies and response times from the pre- and posttraining discrimination task to examine any differences as a function of training and taVNS group. For accuracies (see Figure 5A), there were no differences in accuracies at pretraining between taVNS groups (β = .010, *t* = 0.331, *p* = .742). We also observed no effect of testing time (β = .023, *t* = 1.656, *p* = .106), suggesting that discrimination accuracies did not improve as an effect of training. There were also no significant interactions of testing time and taVNS groups (β = −.004, *t* = −0.188, *p* = .852). Similar findings were observed for response times (see Figure 5B). That is, there were no significant differences in response times as an effect of training (β = .042, *t* = 0.910, *p* = .368) or between taVNS groups (β = −.041, *z* = −0.494, *p* = .623). There was also no significant interaction between testing time and taVNS group on response times (β = −.052, *t* = −0.799, *p* = .429). Together, these results indicate that the ability to accurately discriminate nonnative speech sounds and the speed of discrimination did not change with training or differences in taVNS.

Next, we performed INDSCAL on the inverse of the response time data obtained from the speech discrimination task to understand how participants weighed each perceptual dimension as a function of training and taVNS group. The average weights for each group at each testing time are displayed in Table 3. The group stimulus space of the two-dimensional INDSCAL space is shown in Figure 5C. Dimensions 1 and 2 are interpretively labeled “Relative Pitch” and “Pitch Change,” respectively. We conducted a 2 × 2 mixed ANOVA (taVNS Group × Testing Time) on each dimension. For Dimension 1, we did not observe a significant interaction of group and testing time, *F*(1, 36) = 3.293, *p* = .156, η^2^ = .084. There were also no significant effects of taVNS group, *F*(1, 36) = 3.397, *p* = .148, η^2^ = .086, nor testing time, *F*(1, 36) = 2.068, *p* = .318, η^2^ = .054. For Dimension 2, the ANOVA revealed no significant effects of participant group, *F*(1, 36) = 3.474, *p* = .142, η^2^ = .088; testing time, *F*(1, 36) = 1.754, *p* = .388, η^2^ = .046; nor group by testing time interaction, *F*(1, 36) = 4.072, *p* = .102, η^2^ = .102. These results confirm that participants in both taVNS groups did not differ at pre- and posttraining in their perceptual weighting of the tonal dimensions (see Figure 5D). Crucially, these findings also demonstrate that one group did not have an advantage over the other group at the beginning of the Mandarin tone training task.

**Table 3.** Descriptive statistics of perceptual weights of each dimension from the individual differences scaling analysis.

Variable	taVNS–relative pitch	taVNS–pitch change
Dimension 1 (*M*, *SD*)		
Pretraining	1.031 (0.040)	0.995 (0.036)
Posttraining	1.001 (0.044)	0.998 (0.051)
Dimension 2 (*M*, *SD*)		
Pretraining	0.978 (0.056)	1.029 (0.048)
Posttraining	1.019 (0.055)	1.020 (0.068)

*Note.* taVNS = transcutaneous auricular vagus nerve stimulation.

## Discussion

The goal of this study was to leverage pupillometry to better understand the mechanisms driving taVNS behavioral enhancement. The LC-NE system is hypothesized to underlie VNS behavioral effects, and pupillometry has been established as an index of LC-NE system activity in animals (Aston-Jones & Cohen, 2005; Joshi et al., 2016; Liu et al., 2017; Reimer et al., 2016). Accordingly, we hypothesized that tone categories paired with taVNS would elicit larger tone-evoked pupillary responses than tone categories not paired with taVNS. However, tone-specific pupillary responses did not differ in the presence of taVNS.

One potential reason for the null effects of taVNS on the pupillary response could be that the amplitudes employed in this study may have been insufficient to consistently drive effects. Here, we stimulated at subperceptual threshold levels, such that participants were unaware of when taVNS was being delivered during training. Consequently, all participants in this study were stimulated at amplitudes at or below 1.5 mA, with a median amplitude of 0.6 mA. In comparison, in prior studies that demonstrated pupillary modulation induced by VNS, stimulation was delivered with higher amplitudes than those utilized in this study. For example, Urbin et al. (2021) observed significant pupillary modulation to taVNS, but only at amplitudes greater than 1.0 mA. Similarly, the effects reported by Sharon et al. (2021) were obtained in response to stimulation delivered at levels above the perceptual threshold but below the pain threshold (*M* = 2.20 mA, *SD* = 0.24 mA). Thus, our stimulation amplitudes were overall lower in comparison to previous studies that have demonstrated pupillary modulation in response to taVNS.

Additionally, our results from this study differed from a similar prior study that also paired taVNS with Mandarin tone category learning. Llanos et al. (2020) found that taVNS paired with easier-to-learn relative pitch tones resulted in greater overall learning performance in comparison to a group that received taVNS paired with harder-to-learn pitch change tones. However, we observed no differences in learning performance between taVNS groups. One possibility for this null finding may be that taVNS had no category specific effects but rather widespread effects on learning and pupillary responses. Using our preregistered analyses, we compared data from a group of participants who did not receive taVNS during the task against the two taVNS groups from this study (see Supplemental Material S1). We also found no significant differences in learning performance or pupillary responses between this no taVNS control group and the taVNS groups from this study.

One potential confounding factor in this study that should be noted is our manner of grouping easier-to-learn and harder-to-learn tone categories. We based our groupings off of a large data set that consisted of learning performance across eight similar Mandarin tone category learning studies. In this large data set, the two easiest-to-learn categories were identified as Tone 1 and Tone 3, and the harder-to-learn categories were identified as Tone 2 and Tone 4. Therefore, we had one taVNS group that received stimulation paired with easier-to-learn categories, while the other taVNS group received stimulation paired with harder-to-learn categories. However, even within the easier- and harder-to-learn categories, there is the possibility of variability in learning performance. For instance, Tone 3 tends to be easier than Tone 1, while Tone 4 tends to be easier than Tone 2 (Kirkham et al., 2011). To address this possible confound, our preregistered analyses assessed learning performance for each of the four tone categories individually. We did not observe any differences in learning performance for individual tone categories across taVNS groups. Thus, we can be confident that variability within tone categories did not impact our observed results.

Several factors may have contributed to the differences in findings between this study and the study of Llanos et al. (2020). Notably, the training task in this study was adapted to prioritize pupillometry acquisition, which required changes in trial event timing. To prioritize the pupillary response, trial events were spaced to allow time for the pupil to dilate and return to baseline. Specifically, we implemented a 4-s time window from the onset of the speech stimulus to the response prompt for the category decision, while the prior study prompted the category decision response immediately after the speech stimulus. The extended time between the speech stimulus to the response prompt may have led to the involvement of additional cognitive components that influenced the category decision-making process. For example, a recent pupillometry study demonstrated that individuals with higher working memory have better learning outcomes on the same Mandarin tone category learning task than individuals with lower working memory (McHaney et al., 2021), suggesting that working memory capacity could have been a mediating factor in our current experiment. Additionally, we delayed corrective feedback by 2 s following the response button press. Prior research has demonstrated that delayed feedback leads to poorer learning performance as compared to immediate feedback in speech category learning (Chandrasekaran et al., 2014). As such, it is possible the timing differences in the response prompt and corrective feedback likely contributed to some of the differences in behavioral results between this study and the previous study on taVNS in nonnative speech category learning.

While we did not directly observe pupillary modulation in response to taVNS, we cannot completely rule out contributions from the LC-NE system. Modifications to the procedure for pairing taVNS with auditory stimuli may have also led to differences in the results between this study and the study of Llanos et al. (2020). Here, the taVNS–speech stimulus alignment was adjusted such that taVNS began 50 ms before the onset of the speech stimulus and continued through the entire duration of the stimulus, whereas in the Llanos et al. study, taVNS was delivered beginning at 300 ms prior to the speech stimulus and continued through approximately half (250 ms) of the duration of the speech stimulus. Thus, stimulation in the Llanos et al. study was only delivered during the initial portion of the stimulus that provides relative pitch information (see Figure 1A) but not pitch change information. Conversely, in this study, stimulation lasted for the entire duration of the speech stimulus, covering the acoustic samples that distinguish both relative pitch and pitch change information. It is possible that these subtle changes in taVNS–speech stimulus alignment biased listeners in this study to pay attention to both relative pitch and pitch change information in the speech stimulus. Stimulating the latter half of the stimulus may have biased the listeners in the taVNS–pitch change group to attend to the pitch changes in Tone 2 and Tone 4. Thus, any group specific taVNS enhancement for the taVNS–relative pitch group may have been dampened due to the potential stimulation advantage for the taVNS–pitch change group. Additionally, in line with recent studies on the cortical effects of different taVNS parameters (Schuerman et al., 2021), we used a 30-Hz pulse rate with a 250-μs pulse width, as opposed to a 25-Hz pulse rate and 150-μs pulse width in the Llanos et al. study. Collectively, our results indicate that even subtle changes in taVNS parameters and timing may be critical for behavioral plasticity effects and engaging the LC-NE system. Future studies should investigate optimal taVNS parameters and stimulation intensities to induce behavioral plasticity, particularly using within-subject design.

In addition to our preregistered protocol, we explored the extent to which stimulation amplitude affected learning performance and response times. We found that individuals who were stimulated at lower taVNS amplitudes had better overall learning performance. Notably, the presence or absence of taVNS on a given trial also influenced the rate of learning, that is, individuals had a faster increase in accuracy on trials paired with taVNS than trials without taVNS. Interestingly, the difference in the rate of learning between stimulated and nonstimulated trials was larger for individuals who received taVNS at lower amplitudes. This suggests that taVNS amplitude may influence nonnative speech category learning and behavioral plasticity in a nonmonotonic fashion. These findings support prior research in animals (Borland et al., 2016) and humans (Clark et al., 1999), indicating that VNS amplitudes are an important parameter for inducing behavioral plasticity. As for response times, we observed no differences in the reduction in response times across the training task as a function of stimulation or taVNS amplitude. This null result aligns with prior work, which observed no changes in response times within a single session of Mandarin tone category learning with taVNS (Pandža et al., 2020). However, it should also be noted that there was a 4-s delay from stimulus onset to the response prompt. Response times were time-locked to the onset of the response prompt. Therefore, participants may have already made conscious category decisions in the 4-s period while they were waiting to make the physical button press response. As such, we cannot come to a finite conclusion about the effects of stimulation and taVNS amplitude on response times from current experiment design.

We also did not observe any differences on the discrimination task. Accuracies, response times, and individual variability in cue-weighting did not differ between pre- and posttraining nor as a function of taVNS group. Prior research has demonstrated changes in individual variability in cue-weighting after multiple training sessions across several days (Chandrasekaran et al., 2010). However, pairing taVNS with Mandarin tone learning in a single session did not induce changes in how individuals weigh cues for discrimination. This suggests that there may be some fundamental differences in the mechanisms underlying discrimination versus nonnative speech category learning. Neural representations for nonnative speech categories can emerge in the superior temporal gyrus after a single session of training (Feng et al., 2019), but the emergence of such categorical representations may not be enough to drive differences in discrimination. Furthermore, the fidelity of sensory encoding of nonnative speech categories improves after extensive training over a period of days (Reetzke et al., 2018) but is not improved after a single session of taVNS (Llanos et al., 2020). Therefore, the cue weighting of Mandarin tones in the discrimination task may be driven by the fidelity of sensory encoding and not purely on the emergence of categorical representations. Future studies with extended periods of training sessions could examine how long-term effects of training paired with taVNS impacts discrimination abilities.

A comparison of our exploratory findings with those from a similar Mandarin tone learning pupillometry study (McHaney et al., 2021) indicated that individuals in this study who received high amplitude stimulation performed comparably to individuals who received no stimulation in the McHaney et al. (2021) study. Crucially, individuals who received low amplitude stimulation performed better than those with high amplitude stimulation in this study and those who received no stimulation in the McHaney et al. study (see Supplemental Material S2). These findings align with both human and nonhuman animal studies linking VNS amplitude to behavioral performance and changes in neural activity. For instance, rats receiving VNS at 0.4 mA had enhanced retention performance on an inhibitory avoidance task than those stimulated at 0.2 and 0.8 mA and a control group (Clark et al., 1995, 1998). These findings have been extended to memory performance in humans as well, where lower VNS amplitudes were related to greater word recognition scores (Clark et al., 1999). The amplitude of taVNS has also been found to modulate neural activity, with increases in cortical theta band power found during moderate amplitude stimulation and decreases in theta band power found during higher amplitude stimulation (Schuerman et al., 2021).

Prior work has shown that VNS amplitude can modulate cortical plasticity. For instance, VNS paired with the presentation of a 9000-Hz tone, cortical map plasticity was found to vary as a function of amplitude (Borland et al., 2016). Rats that received VNS at moderate amplitudes of 0.4–0.8 mA showed a greater increase in the number of cortical neurons tuned to that frequency than rats that received VNS at higher amplitudes of 1.2–1.6 mA. Borland et al. (2016) posit that low-to-moderate amplitude VNS facilitates neuroplasticity, while higher VNS amplitudes may trigger inhibitory mechanisms that prevent plasticity from occurring. In the context of this study, it could be that the mechanism of action of VNS on behavioral plasticity differs from the mechanism that influences pupillary changes associated with VNS. For instance, cholinergic activity has been implicated in modulating sensory plasticity (Engineer et al., 2011; Kilgard & Merzenich, 1998). Some invasive VNS studies have demonstrated cholinergic-induced plasticity in the primary auditory cortex at stimulation amplitudes below 1.6 mA (Borland et al., 2016; Engineer et al., 2015), which aligns with the range of stimulation amplitudes from this study.

VNS amplitudes may also be critical for maintaining an optimal balance between LC phasic and tonic modes to optimize behavioral plasticity and for activating the neural circuits that drive plasticity. Arousal states and behavioral performance are driven by the balance between LC phasic and tonic modes (Aston-Jones & Cohen, 2005). Higher tonic LC activity is associated with an increase in global NE levels and a reduction in behavior, while phasic activity is associated with task-oriented behaviors to optimize performance. Therefore, we posit that higher taVNS amplitudes may lead to an overrelease of NE that (a) increases tonic activity, (b) increases pupil dilation, (c) inhibits cholinergic activity, and (d) reduces behavioral performance and plasticity. This suggests that lower taVNS amplitudes may facilitate behavioral plasticity during nonnative speech category learning due to a better balance between LC phasic and tonic modes and optimal cholinergic activity, with no detectable changes in pupil dilation, while pupillary responses may only serve as a marker of taVNS efficacy at higher stimulation amplitudes. In summary, low-amplitude taVNS may be key for behavioral plasticity during nonnative speech category learning, but these parameters may not be enough to modulate noninvasive physiological biomarkers of LC-NE activity and taVNS efficacy, such as the pupillary response. Future research should continue to explore the role of stimulation amplitude in taVNS efficacy and the possibility of differing mechanisms between plasticity and pupillary modulation.

In conclusion, our results indicate that taVNS does not modulate pupillary responses during nonnative speech category learning. Our findings do not support pupillary responses as a reliable marker for subthreshold taVNS efficacy. Before further advancements of taVNS applications in humans for clinical and nonclinical purposes can occur, the stimulation parameter space needs to be comprehensively assayed to ensure consistent effects and reliable markers. However, we did observe preliminary results that suggest the magnitude of taVNS amplitude may modulate learning performance, but additional research is needed to validate these amplitude effects. Collectively, the results from this study indicate that, in contrast to the often clear and consistent VNS effects on the pupillary response reported in nonhuman animal literature, identifying behavioral and physiological correlates of taVNS efficacy in humans is a complex enterprise.

## Data Availability Statement

The datasets generated during and/or analyzed during the current study are available in the Open Science Framework repository and can be accessed at https://osf.io/zw9m7.

## Supplementary Material

10.1044/2023_JSLHR-22-00596SMS1 Supplemental Material S1 Pre-registered analyses including a no-taVNS control group.

10.1044/2023_JSLHR-22-00596SMS2 Supplemental Material S2 Accuracy comparison with McHaney et al., 2021.
