# Supplementary material for: Transcutaneous Auricular Vagus Nerve Stimulation Modulates Performance but Not Pupil Size During Nonnative Speech Category Learning
Source: J Speech Lang Hear Res. 2023 Aug 31;66(10):3825–43. doi: 10.1044/2023_JSLHR-22-00596 (PMC12379271; doi:10.1044/2023_JSLHR-22-00596)
Supplement: Supplemental Material S1 [file JSLHR-66-3825-s001.pdf]

Supplemental Material S1. Pre-registered analyses including a no-taVNS control group.

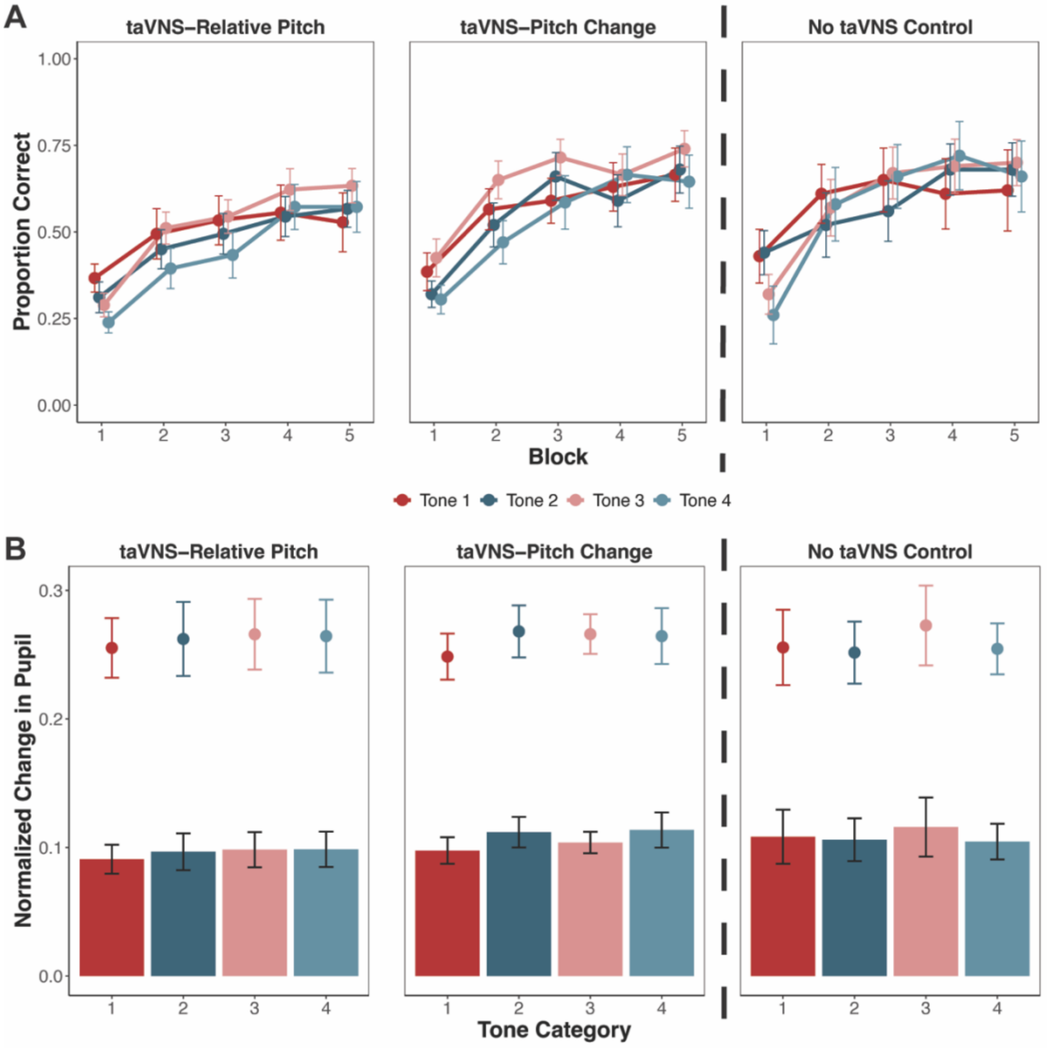

Figure S1. Pre-registered analyses including a no-taVNS control group. An additional 10 participants were recruited into a no-taVNS control group to examine the effects of taVNS on the accuracies and pupillary responses relative to those who received no taVNS intervention.

A) We added the no-taVNS control group to the original preregistered binomial generalized linear mixed effects model analysis to examine differences in accuracies based on tone categories. The outcome variable of the model was trial-by-trial accuracy (correct, incorrect) for each participant. The model included fixed effects of trial, tone category, and group, as well as all 2-way and 3-way interactions between fixed effects. The model included a maximal random effect structure with random slopes of subject per trial, item per trial, and a random slope for the interaction between subject and tone category per trial: $Accuracy \sim trial * tone\ category * group + (trial \mid subject) + (trial \mid subject{:}\ tone\ category) + (1 \mid item)$. This model provided a significantly lower AIC value than models with only a random intercept of trial per subject or intercepts of trial per subject and item ($\chi^2(3) = 161.091$, $p < .001$; $AIC_{(trial|subject)} = 11{,}102.830$; $AIC_{(trial|subject)+(1|wav)} = 10{,}900.270$; $AIC_{final} = 10{,}745.18$). We did not observe any significant effects of group (*p*s > .05)

or interactions of trial and group (*p*s > .05), indicating that participant groups did not differ in their overall accuracy nor their trial-by-trial increase in accuracies. Multiple pairwise comparisons also did not reveal any significant effects of tone category, interactions of trial and tone category, nor the interactions of trial, group, and tone category (*p*s > .05). These results suggest that taVNS did not have any tone-specific or group enhancements on learning accuracy.

B) Separate linear mixed-effects models were estimated to examine mean and max pupillary dilation time-locked to the speech stimuli during the Mandarin tone category learning task. Fixed effects included main effects of tone category, taVNS group, and the interaction between tone and taVNS group. A model containing a random intercept of subject and random slope of taVNS group per item provided a singular fit, thus the final model included only a random intercept of subject: $Pupil \sim tone\ category * group\ + (1 \mid subject)$. We did not observe a significant effect of tone category (*p*s > .05), groups (*p*s > .05), nor a significant interaction between tone category and groups (*p*s > .05). For the maximum pupillary response, we also did not observe any significant effects of tone category (*p*s > .05), groups (*p*s > .05) or interactions between tone category and groups (*p*s > .05). Taken together, these results suggest that taVNS did not have any tone-specific effects on the average pupillary response and the peak dilation size between taVNS groups and the no taVNS control group.
