# Supplementary material for: Transcutaneous Auricular Vagus Nerve Stimulation Modulates Performance but Not Pupil Size During Nonnative Speech Category Learning
Source: J Speech Lang Hear Res. 2023 Aug 31;66(10):3825–43. doi: 10.1044/2023_JSLHR-22-00596 (PMC12379271; doi:10.1044/2023_JSLHR-22-00596)
Supplement: Supplemental Material S2 [file JSLHR-66-3825-s002.pdf]

Supplemental Material S2. Accuracy comparison with McHaney et al., 2021.

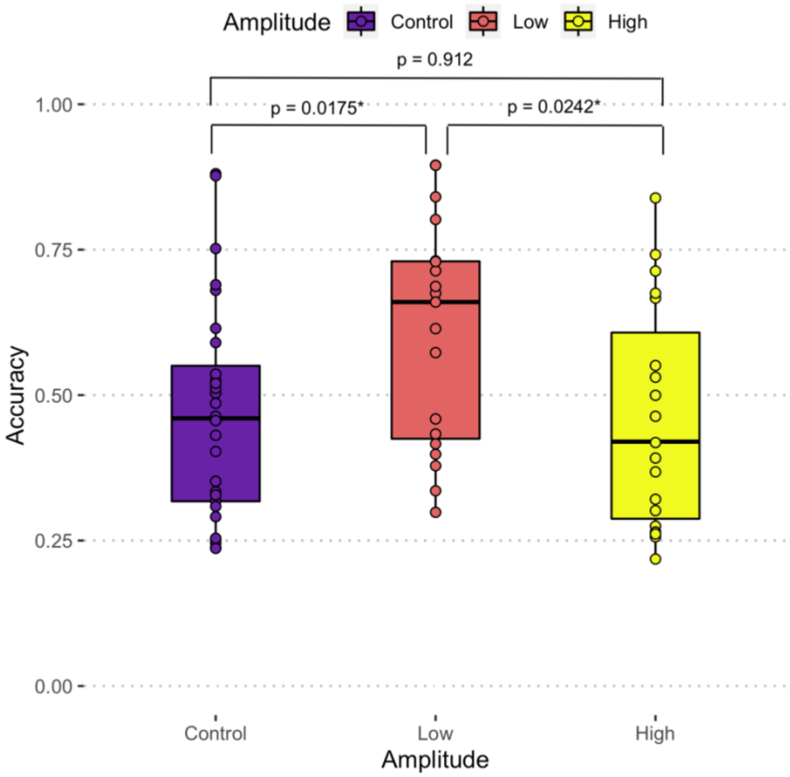

Figure S2. Accuracy comparison with McHaney et al., 2021. Boxplots indicate median and interquartile range for "Control" participants (purple; data adapted from McHaney et al., 2021), participants in the current study who received Low amplitude stimulation (red; min = 0.1, max = 0.6, mean = 0.34) or High amplitude stimulation (yellow; min = 0.7, max = 1.5, mean = 1.08). To facilitate comparison with McHaney et al., 2021, accuracy was only calculated over training blocks (generalization block excluded). A one-way ANOVA on stimulation amplitude revealed that groups differed by accuracy ($F(2) = 3.83$, p = 0.027). Post-hoc t-tests for between group contrasts indicated that participants in the Low amplitude group tended to respond more accurately during training compared to participants in the High amplitude group ($p = 0.0242$) or participants in the Control group who did not receive stimulation ($p = 0.0175$). The Control group and the High amplitude groups did not differ significantly with regards to accuracy ($p = 0.912$).
